# Optimized Sensor Network and Multi-Agent Decision Support for Smart Traffic Light Management

**DOI:** 10.3390/s18020435

**Published:** 2018-02-02

**Authors:** Luis Cruz-Piris, Diego Rivera, Susel Fernandez, Ivan Marsa-Maestre

**Affiliations:** Departamento de Automática, Escuela Politécnica Superior, Universidad de Alcalá, 28805 Alcalá de Henares, Madrid, Spain; diego.rivera@uah.es (D.R.); susel.fernandez@uah.es (S.F.); ivan.marsa@uah.es (I.M.-M.)

**Keywords:** sensor networks, optimized sensor deployment, multi-agents system, intelligent transportation system, smart cities, traffic simulations, traffic light management

## Abstract

One of the biggest challenges in modern societies is to solve vehicular traffic problems. Sensor networks in traffic environments have contributed to improving the decision-making process of Intelligent Transportation Systems. However, one of the limiting factors for the effectiveness of these systems is in the deployment of sensors to provide accurate information about the traffic. Our proposal is using the centrality measurement of a graph as a base to locate the best locations for sensor installation in a traffic network. After integrating these sensors in a simulation scenario, we define a Multi-Agent Systems composed of three types of agents: traffic light management agents, traffic jam detection agents, and agents that control the traffic lights at an intersection. The ultimate goal of these Multi-Agent Systems is to improve the trip duration for vehicles in the network. To validate our solution, we have developed the needed elements for modelling the sensors and agents in the simulation environment. We have carried out experiments using the Simulation of Urban MObility (SUMO) traffic simulator and the Travel and Activity PAtterns Simulation (TAPAS) Cologne traffic scenario. The obtained results show that our proposal allows to reduce the sensor network while still obtaining relevant information to have a global view of the environment. Finally, regarding the Multi-Agent Systems, we have carried out experiments that show that our proposal is able to improve other existing solutions such as conventional traffic light management systems (static or dynamic) in terms of reduction of vehicle trip duration and reduction of the message exchange overhead in the sensor network.

## 1. Introduction

Intelligent Transportation Systems (ITS) is a concept that includes all the technological solutions and research proposals which, through an intelligent systems approach, aim to solve and improve the issues related with traffic management. The applications of ITS are very diverse, as this kind of system encompasses all of the elements in the road (from the infrastructure to vehicles and users).

This paper is an extended version of the work presented in JITEL 2017 [1], where we proposed the use of a Multi-Agent System (MAS) composed of multi-behavioral agents to optimize the management of traffic lights. This work led us to focus our research on obtaining an optimal set of sensor locations in the network used to provide data to the MAS. The results of this work contribute to this specific goal.

The sensors distributed across the traffic network generate the knowledge base in which the ITS relies on for the decision-making process. Traffic sensor technologies have noticeably improved in the last years, but, although there are some sensors not dependent on the roadway infrastructure (for instance, the Global Positioning System (GPS) installed in Smartphones), many of them still require an installation process and maintenance, which is traduced at a high cost. This cost limits the number of sensors that can be deployed in these environments. Therefore, the correct selection of sensor location in a traffic sensor network is crucial for guaranteeing the efficiency and functionality of the network.

The intelligent management of traffic lights is a common method used to intervene in the events produced in these scenarios. One of the biggest challenges in the traffic light management systems is to be able to process all the information that is produced in their environment and make effective decisions using it. MAS can provide important advantages in solving this kind of challenge, as they offer the possibility of dividing the problem into smaller sub-problems, and therefore improve scalability and efficiency of the system. In Section 2, we show a selection of works in sensor locations in sensor networks, studies on the application of graph theory for traffic networks, and ITS based on MAS.

**Figure 1:**
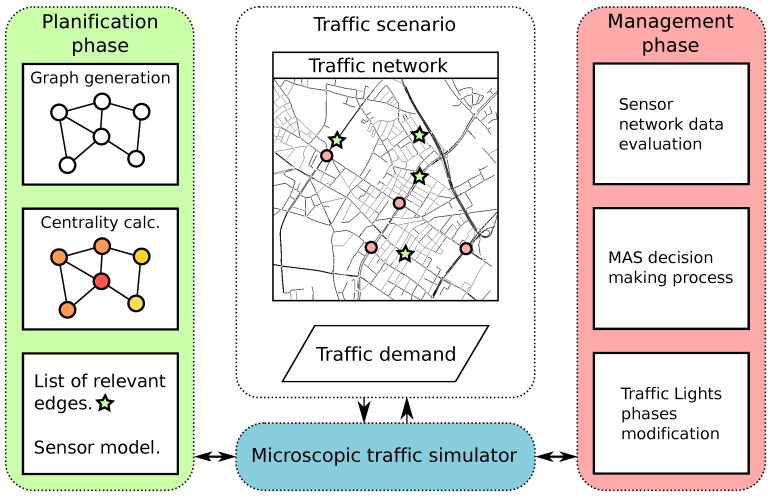
Overview of the proposed system.

Our proposal can be divided into two main phases as shown in Figure 1. The first one, planification phase, is focused on defining a methodology to obtain a list of possible sensor locations ordered by their relevance. This list will be based on a study of the centrality (indicator of the importance of each node in a graph) for the graph determined by the traffic network and historical data of traffic demand (this phase is detailed in Section 3). Once the network is optimized by placing sensors in the more relevant locations, we define an MAS to manage the phases of the traffic lights in the network (management phase). In our proposed MAS, we define agents for intersection traffic light management and coordination between intersections (which is detailed in Section 4).

Most of the current proposals (as shown in Section 2) for the improvement of sensor locations in sensor networks are based on using information from the network itself (for instance, historical data from traffic flows). One of the novelties of our method when compared with others is that we use indicators that are only based on the network topology (once it is transformed into a graph) for our proposed algorithm. These indicators allow us to obtain near-optimal locations, which can also be improved by using real traffic data.

The validation of the proposed solution is performed using a microscopic traffic simulator and a scenario based on real-life data from the city of Cologne in Germany (Section 5). Then, we discuss the obtained results both from the planification phase and the management phase of the proposal (Section 6).

## 2. Related Work

There are many factors involved in the correct circulation of vehicles. In fact, problems related to vehicle traffic are widely studied, as it is one of the most relevant challenges in modern societies. ITS are used with goals as diverse as improving toll collection [2,3], data collection from highways [4,5] and vehicles [6], emergency vehicles preemption [7], improving the transit signal priority [8] or other traffic management systems [9]. Using the data from the traffic sensor networks, ITS can offer solutions to solve many traffic related problems [10] such as traffic jams in certain zones or time slots.

In this section, we will comment the related work in sensor networks used in traffic scenarios, specifically those focused on sensor location, intelligent traffic light management, and the use of MAS in distributed traffic problems.

There are some works specifically focused on using sensor networks for road traffic management. In the paper in [11], the authors characterize and discuss probable topologies of these networks. The aim of the work is to provide network models that can be used to evaluate protocols and algorithms using realistic scenarios in place of generic random graphs. They deploy such networks over 52 city maps extracted from OpenStreetMap and characterize the resulting graphs, with focus on the connectivity aspects (degree distribution and connected components). The use of the sensor networks for measuring traffic volume, waiting time, vehicle density and other factors, which can be used as parameters for an algorithm to adapt the traffic light phases length is also approached in [12]. There is also a proposal for using sensor networks to feed an algorithm for flow control and congestion avoidance in traffic networks [13]. They improve the average waiting time and queue length both in isolated intersections and multiple intersections.

In [14], the authors present an ontology-based architecture to improve the driving environment through a traffic sensor network. The system performs different tasks in an automated way such as the traffic light management that includes the adjustment of the duration and intensity of the light, considering the traffic flow and the weather conditions. Another contribution in this topic can be found in [15], where they use fuzzy logic controllers to manage dynamically the traffic light phases, using also as input the information of a wireless sensor network.

In the literature, there is a large number of works related with the optimal location of sensors in Wireless Sensor Networks (WSNs). Many of these works are focused specifically on finding the best location of the sensors used in vehicular traffic networks. Some of these proposals are based on the use of Evolutionary Algorithms (EA) to obtain the optimal sensor locations. Kulkarni and Venayagamoorthy [16] show a study on the use of Particle Swarm Optimization (PSO) in WSN to optimize the number and location of sensors in terms of coverage, connectivity and energy efficiency. This kind of optimization technique is popular due to its simplicity, high quality of solutions, fast convergence, and insignificant computational burden. The drawbacks of using PSO are that they require large amounts of memory and that they might not be suitable for real-time applications. Based on using historical vehicular traffic data, Shan and Zhu [17] propose a methodology to locate camera sensors to estimate traffic in real time. As a data source, they use real GPS data from more than 8000 taxis. They transform the problem in a graph theory problem and then they propose a method based on a greedy algorithm to obtain an optimal result. This proposal obtains good results when compared with random sensor positioning or a positioning based on the most important roads in the network. The experiments lack validation through a simulation system, which could bring the solution closer to its real-life implementation.

Another approach to this problem is the one defined as the observability problem [18], which is based on determining the number of observed traffic flows and installing the required instruments to make it possible. The problem is classified in four sub-categories: link flow observability, origin-destination (O-D) flow observability, route flow observability and general case flow observability. Focusing on the first sub-category, which consists of determining which subset of link flows can be calculated in terms of another subset of links flows, there is a previous study by Hu et al. [19], where they work on the Network Sensor Location Problem (NSLP), which addresses the same challenges. NSLP has been used to determine the minimum number of sensor locations or the optimal locations for a given number of sensors, in order to estimate the O-D demand from historical data.

The methods we have described are focused on obtaining traffic flows in a network using information from a limited subset of sensors. Although the results are good, the complexity of the systems is high, and most of them require an a priori knowledge of the historical traffic flows (for instance, an estimation of O-D matrices). Moreover, the location of sensors that would benefit the construction of O-D matrices do not necessarily coincide with the areas in the network with a high traffic density. These areas are more interesting for monitoring to detect real-time traffic problems such as traffic jams.

Regarding the traffic light management, it is possible to find many works where EA are used to investigate the potential of these types of algorithms for the optimization of traffic light controllers. For instance, in [20], they use a Genetic Algorithm (GA), one of the most popular algorithms of this category. They conclude that, for solving these types of problems, it is useful to use evolution strategies. In [21], authors show the use of an iterative optimization algorithm (specifically a PSO algorithm) to find successful cycle programs of traffic lights. They validate their proposal using the Simulation of Urban MObility (SUMO) microscopic traffic simulator, obtaining an improvement in terms of total trip times and number of vehicles that arrive at their destination in a predefined simulation time.

Ref. [22] proposes a real-time adaptive lighting scheme, which detects the presence of vehicles and pedestrians and dynamically adjusts their brightness to the optimal level. This improves the energy efficiency of street lighting and its usefulness. The proposed scheme is simulated using an environment modelling a road network, users, and a networked communication system and considers a real streetlight topology from a residential area.

In ITS, the use of MAS is also common, given that the geographically distributed nature of the traffic elements, the dynamic environment, and the strong interaction between these elements can be easily approached using a distributed architecture such agent-based technologies [23]. For instance, in [24], there is a thorough review on agent-based technology for traffic, grouping the works in two main categories: modelling and simulation, and control and management. The paper in [25] also reviews works that use agent technologies to improve traffic related problems.

Some other works use the information generated by sensor networks to feed MAS focused on the traffic light management. In [26], the authors combine the information from sensors with an MAS to provide an adaptive management.

The work presented in [27] defines the generic elements that should compose the MAS for urban signal control, while Ref. [28] proposes a hierarchical multi-agent architecture that includes the internal description of the system. In both works, they demonstrate the viability of using MAS applied to traffic light management.

The ACTAM (Adaptive and Cooperative Traffic light Agent Model) system [29] aims to reduce the traffic congestion in urban roads by using a complete agent-based system that synchronizes and improves the efficiency of traffic management in cities. Unfortunately, it has only been validated in a small traffic network with about 30 intersections, which is far from a real-life scenario. Furthermore, the results are only compared with fixed-time signal control, and are not compared with other available management systems like actuated control traffic.

In Ref. [30], CARTESIUS, another architecture based on agents is proposed. The goal of this architecture is to provide a cooperative system to allow the collaboration between systems managed by different authorities to solve congestion problems. Analogously, Ref. [31] proposes an MAS based on mobile agents for traffic management and control, and, in [32], the authors propose using negotiation techniques between agents to optimize vehicular routes, based on the contents of a traffic data matrix.

## 3. Design and Optimization of a Sensor Network for ITS

In Section 2, we have shown the main current sensor architectures applied to ITS. In this section, we first show a classification of the sensors used in these environments and we define how we model them in a microscopic traffic simulator. Then, we propose a methodology for the optimal location of the sensors across the network. We aim to reduce as much as possible the number of messages exchanged by them, while maintaining enough information for a complete view of the scenario.

### 3.1. ITS Sensor Classification and Simulation Models

ITS can improve by using information offered by different types of sensors. The sensors in charge of measuring physical parameters such as temperature, humidity, wind velocity, luminosity, etc. can aid the intelligent systems to be more efficient. In this section, we focus on those sensors that can obtain information from the traffic network itself, considering the network and the vehicles using it.

A first classification can be made between sensors that offer direct traffic information: it is possible to differentiate between those that require the installation of equipment in the vehicles (e.g., GPS) and those that can be considered autonomous [33]. In addition, these autonomous sensors can be classified in those that are embedded in the pavement of the roadway and those that are embedded above or alongside the roadway [34].

In Table 1, we are showing a selection of these sensors.

Modern traffic simulators feature the possibility of using some of these sensors. It is important that these sensors behave as closely as possible to their real-life counterparts. The data output of SUMO can offer information about what has happened in each simulation from the vehicle or edge point of view. Therefore, it is possible to model the sensor behavior using these features. This information can be analyzed once the simulation has ended or during the simulation time. The communication between both modules is performed using the Traffic Control Interface (TraCI) tool [35], included in the SUMO package. This tool provides a Transmission Control Protocol (TCP) based client/server architecture that allows to control and modify the SUMO simulations through an external application.

**Figure 2:**
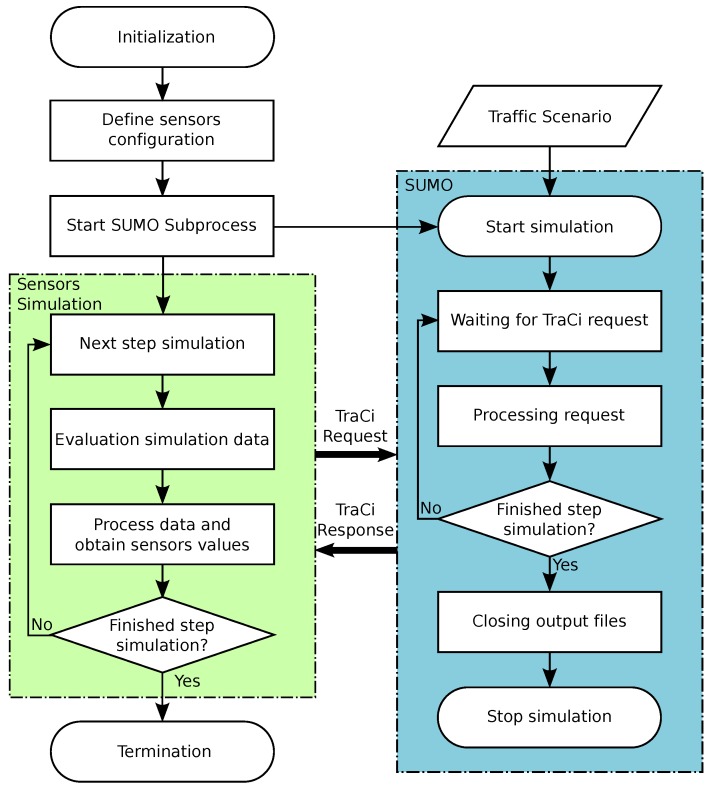
Block diagram of the sensor modelling and simulation platform.

In Figure 2, we show the communications between the sensor modelling process and the traffic simulator.

The main steps followed during system execution are:

1. System initialization: The application reads the data from the scenario, initializes the sensor configuration, including the definition of its features, and launches a subprocess that starts the SUMO simulator.
2. Loop until end of the simulation: There is a parameter in the application configuration that states the duration in seconds of each simulation step. The sensor simulation module will perform the following tasks including requests to the simulation module (via TraCI):
  (a) Requests one step simulation and then waits for the end of the step,
  (b) Requests the current parameters of each vehicle that was active in that step of simulation and the edge information,
  (c) Processes the information, and models it for the specific sensors,
  (d) Returns the values generated for the sensor,
3. End of the simulation.

Depending on the specific traffic sensor, the modelling process can be complex. For instance, GPS sensor modelling can be performed by obtaining the relative position and instant velocity value from the simulation data. This value can be modified by applying a correction factor to add the possible error introduced by GPS real systems accuracy (e.g., in GPS-enabled smartphones the typical accuracy is within a 4.9 m radius under open sky [36]).

Other sensors, such as video cameras, need a previous study to define its characteristics. To carry out this study, we need the camera installation height, its focal length, and its inclination angle, among other parameters. Typical values for a camera installed in a roadway could be 8 mm of focal length and 12.2 m of height. Using these values, we could obtain a minimum distance value of 15.4 m and a maximum of 139.4 m, and values from 15.7 m to 112 m in width [33]. According to this, we can model the simulated camera by obtaining the number of vehicles that are located within the range of vision of the camera starting from the camera position (in the example above, a maximum of 124 m from the camera, and with a width of 15.7 m). As with GPS simulated sensors, a correction factor can be added to model the possible camera errors.

### 3.2. Sensor Location Proposal

The vehicle embedded sensors depend on the technology available in each vehicle and the driver’s preferences. Although the price of the sensor is lowering for some of them, it is still a high cost, and this is especially true for the infrastructure needed for some of them. This means that it is an essential issue to position the sensors wisely across the network.

In a traffic scenario, we must be able to obtain a global view of what is happening at each time. Depending on the type of sensor, we can get data that is related (e.g., traffic density data in some place across the network and the weather conditions of that same place).

In a traffic network, there are certain spots that will be revealed as especially relevant, as they will be important in a later analysis that will feed the ITS and help in the decision-making process. Detecting those spots will allow us to position in them autonomous sensors. These sensors will offer accurate and constant information about their surroundings, without depending on the information from vehicle embedded sensors, which will lead to a global improvement in the data quality.

These relevant spots can be calculated using historical data of the vehicular traffic flows, as we can select those where the highest number of coincident traffic flows is shown. Unfortunately, the complexity of obtaining a reliable traffic model makes necessary to design methodologies to obtain the spots using different parameters.

Our proposal is abstracting the traffic network as a directed graph, and then study its centrality. Through this study, it is possible to obtain a ranking of the most relevant edges. Then, we can combine this ranking with a ranking obtained from traffic historical data, and then obtain a list of spots where the sensors would provide the most useful information.

The volume of vehicles in an edge depends on the traffic demand of the edge and on the ability of the edge to be a connector based on its location in the road, that is, in its betweenness centrality. There are studies that establish a relationship between the traffic flows and the betweenness centrality values of a graph [37]. This centrality of an edge is “proportional to the number of shortest paths between all pairs of nodes passing through it” [38] and can be measured by averaging over each pair of nodes and following the shortest path to the destination. Consequently, we have chosen the betweenness centrality to obtain the ranking of relevant edges in the network. In Equation (1), we show the betweenness centrality calculation for one node, as proposed in [39]:

(1) $$c_{B}\left(v_{i}\right)=\sum_{s,t\in V}\frac{\sigma (s,t|v_{i})}{\sigma (s,t)},$$

where *V* the set of nodes from a directed graph, σ(s,t) is the number of shortest paths between *s* (source node) and *t* (target node), and $\sigma (s,t|v_{i})$ is the number of those paths passing through a node $v_{i}$ different from *s* and *t*.

The methodology we propose is based on calculating the following data structures:

1. $L_{c}$: List of edges ordered by their centrality value. Obtained following the next steps:
  (a) Transformation of the traffic network in a directed graph (*G*):
    (2) G(V,E),
    where *V* is the set of network nodes and *E* is the set of network edges.
  (b) Calculation of the betweenness centrality of *G*. There are implementations that allow to calculate the betweenness centrality of the edges of the graph [40]. Another option would be to transform the directed graph *G* into a Line-graph ($G_{LG}$), in which the nodes are constituted by the edges of *G* and the edges are the possible turns from one edge to other in the original graph [41]:
    (3) $$G_{LG}\left(V_{L},E_{L}\right),$$
    (4) $$V_{L}=E,$$
    where $V_{L}$ is *E* from *G* and $E_{L}$ is the set composed by the possible turns from one edge to other in *G*. We obtain the centrality measurement of $G_{LG}$ in the set $S_{c}$:
    (5) $$S_{c}=\overset{n}{\underset{i=0}{⋃}}\left\{\left(v_{L_{i}},c_{B}\left(v_{L_{i}}\right)\right)\right\}.$$
    Applying Equation (4) in Equation (5):
    (6) $$S_{c}=\overset{n}{\underset{i=0}{⋃}}\left\{\left(e_{i},c_{B}\left(v_{L_{i}}\right)\right)\right\},$$
    where $e_{i}$ is each of the *n* elements in *E* (set of edges of the original graph *G*) and $c_{B}\left(v_{L_{i}}\right)$ is the centrality value of $e_{i}$.
  (c) $L_{c}$ is obtained from the ordination of the set $S_{c}$ from the highest to the lowest centrality value, and the normalization of the centrality values over 1.
2. $L_{d}$: List of edges ordered by their historical occupation level. Obtained following the next steps:
  (a) Obtaining of the historical traffic values. These values might be already grouped or might be a traffic model that should be simulated to obtain numerical values. We define $S_{d}$ as:
    (7) $$S_{d}=\overset{n}{\underset{i=0}{⋃}}\left\{\left(e_{i},v_{d_{i}}\right)\right\},$$
    where $e_{i}$ is each of the *n* elements in *E* and $v_{d_{i}}$ is the historical occupation value for $e_{i}$.
  (b) $L_{d}$ is obtained from the ordination of the set $S_{d}$ from the highest to the lowest historical occupation value, and the normalization of the historical occupation values over 1.
3. $L_{p}$: List obtained using the combination of $L_{c}$ and $L_{d}$, and using a reliability factor for the historical values (α):
  (8) $$L_{p}=\left(1-\alpha \right)L_{c}+\alpha L_{d}.$$
  (a) α is a value between 0 and 1 that measures the reliability level of the historical data, where 1 would be the closest possible value to the real-life one.
  (b) Ordination of $L_{p}$ from the highest to the lowest value.

Once this process is finished, $L_{p}$ offers an ordered list of the most relevant edges in the network, where we can position the sensors to obtain better and more reliable information.

In Algorithm 1, we show the pseudo-code of the calculation of the $L_{p}$ list to provide a more detailed view of this process. The algorithm takes a graph G(V,E) as input, and returns the $L_{p}$ list as defined above. The first part of the code generates the line-graph ($G_{LG}\left(V_{L},E_{L}\right)$) used for the calculation of the centrality values. The nodes of this graph ($V_{L}$) are the edges of *G* (line 5), and the edge set ($E_{L}$) is composed by the list of possible turns from one edge to the other in *G*, as it is shown in lines 6 to 11.

Once the line-graph is generated, it is used for the calculation of the $L_{c}$ list, which is composed of tuples of edges and betweenness centrality values for those same edges ($e_{i},c_{B}\left(v_{L_{i}}\right))$. For this calculation, we use an implementation of the betweenness centrality, which, in our pseudo-code, is represented by the function *calculate_centrality* (line 13). The result set returned by the centrality function is ordered by the centrality values and normalized in the list $L_{c}$ (lines 14 and 15).

Additionally, we calculate the $L_{d}$ list, which is the result of formatting the historical data *D* into a set of tuples of edges and values $\left(e_{i},v_{d_{i}}\right)$, and its ordination by the values $v_{d_{i}}$ and normalization (lines 17 to 19).

Finally, for each edge, we calculate a combined value composed of the sum of the corresponding centrality value ($c_{B}\left(v_{L_{i}}\right)$) and historical value ($v_{d_{i}}$) using the reliability factor α to adjust which value will weigh more. The resultant set is ordered by this combined value, returning the desired $L_{p}$ list. This is shown in lines 21 to 25.


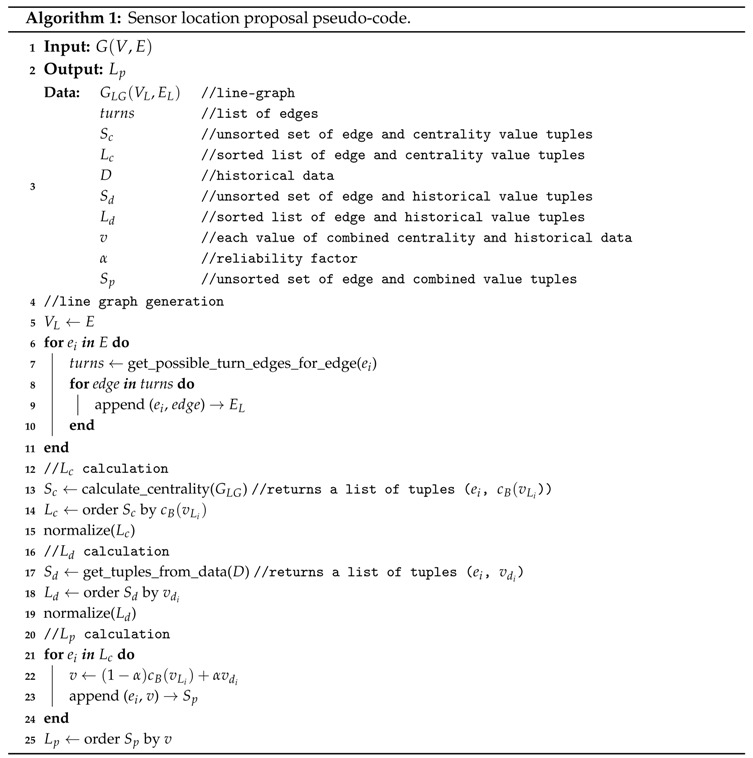


## 4. Multi-Agent System for Intelligent Traffic Light Management

Once we have an optimized sensor network offering us a reliable data source from the traffic scenario, we are using it to feed an MAS for traffic light management. This system’s goal is to reduce the duration of vehicle trips by modifying the intervals of the traffic lights (phases) in a traffic network.

In the following sections, we are going to describe the types of agents involved in the system and the general operation of the system itself.

### 4.1. Agents

In this section, we describe each one of the three types of agents involved in the system. For each agent, we include its description, where it is located in a traffic scenario, and its behavior in the system.

#### 4.1.1. “TLAgent” Traffic Light Management Agent

- **Description**: This kind of agent is defined for the traffic light phases’ changing management. It will be aware of the other agents of the same type in the intersection.
- **Location**: Each traffic light in each intersection of the network will be provided with one of these agents.
- **Behavior**: In an initial state, the traffic light changes will follow a static predefined pattern. Once the agent receives a message indicating a change, it will reconfigure the phases accordingly. The changes will always be performed within a given maximum and minimum time thresholds.

#### 4.1.2. “TJamAgent” Traffic Jam Detection Agent

- **Description**: These agents are in charge of obtaining data from their environment and then making a decision about the traffic state, with the ultimate goal of communicating a possible traffic jam in their surroundings.
- **Location**: These agents are deployed across the sensor network. They can be located both in sensors installed in the network or aboard sensors embedded in the vehicles that transit through it.
- **Behavior**: Agents located in vehicles will base their decision on the data provided by sensors (specifically, the geographic position and instantaneous velocity of the vehicle). A threshold will be defined for this data, and once the sensors provide higher values than expected for a period, the agent will consider a traffic jam situation and notify it. The sensors installed in the network will make the decision using the same mechanism, but relying on different types of data depending on the type of sensor installed.

#### 4.1.3. “IntersectionAgent”

- **Description**: These agents are the ones that decide how the traffic lights of an intersection should be changed. They receive messages from the TLAgents near them, and then decide which traffic flow in the intersection should be prioritized.
- **Location**: They can be located anywhere within the communications coverage of the TLAgents of each intersection.
- **Behavior**: Using the current state of the TLAgents they control (the specific number of agents will depend on the number of lanes ending in the intersection) and information from TJamAgents, they will perform a decision-making process based on a state machine. The decisions will be sent again to the TLAgents. These agents are also communicating between them and they can operate in two modes regarding these communications:
  - **Altruistic or Collaborative**: This mode allow agents to listen to requests from other agents of the same type. The information given by the other IntersectionAgents will be added to the information obtained from the TJamAgents to make a more informed decision on how to prioritize flows.
  - **Selfish or Isolated**: The agents enter this mode when they determine that the zone that they are managing is much too congested and therefore they should make their own decisions instead of pursuing a global cooperative goal. Once in this mode, the agents will ignore any message from other IntersectionAgents until the congestion is lower or a defined timer expires. This timer is set to avoid system blocking if the agents stay too much time in this mode.

### 4.2. MAS Behaviour

From the agent definition shown in Section 4.1, the MAS is able to dynamically manage the traffic lights phases from a traffic scenario. The MAS is able to modify its general behavior to react to traffic jam situations, which can result in the blocking of vehicles for extended periods of time.

**Figure 3:**
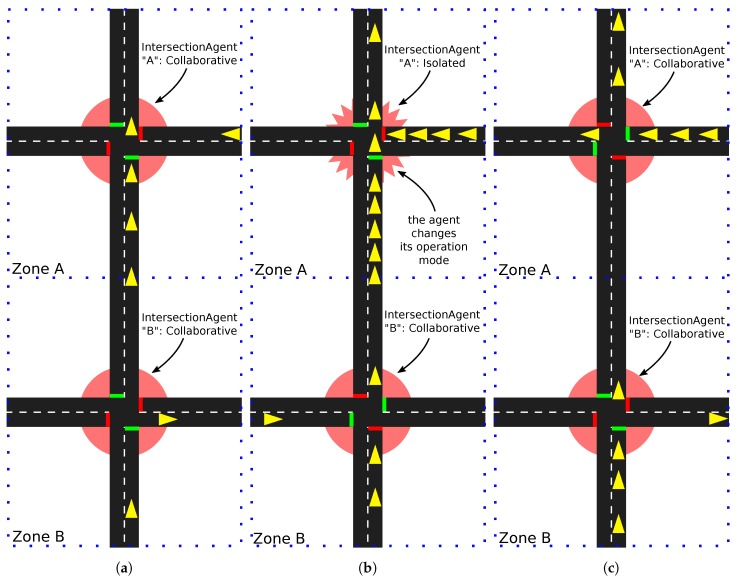
Multi-Agent System operation use case. (**a**) initial state; (**b**) zone “A” congested; (**c**) zone “A” normal flow.

In this section, we are going to propose an example use case composed by a network with two intersections. In this scenario, we are forcing a congestion situation and then show how the agents change their behavior to solve the issue. The different states of the scenario during the system operation are shown in Figure 3. The three types of agents are represented in the figure using red or green lines (TLAgents), yellow triangles (TJamAgents in vehicles) and red areas (IntersectionAgents). In addition, we consider all traffic flows going from the left or the right in the figures are “horizontal flows”, and all traffic going from the bottom or the top as “vertical flows”.

1. **Initial state** (Figure 3a): Both IntersectionAgents (“A” and “B”) will begin their operations in *altruistic* mode. This means that they will exchange information to help each other.
  The vehicles in the vertical traffic flow inside the zone “A” circulate at a speed below the optimal in that road. Therefore, the TJamAgent included in each one, will report a possible traffic jam situation to IntersectionAgent of the zone “A”.
  Once the IntersectionAgent collects the information from the TJamAgents in its zone, it starts the decision-making process and finally decides to prioritize the vertical traffic flow. The actions performed by the agent are: sending the new phases configuration to the TLAgents in the intersection, and advertising the changes to the nearby IntersectionAgents (in this case, the agent in zone“B”) to maintain the synchronization of the adjacent intersections.
2. **Zone “A” Congested** (Figure 3b): Once an IntersectionAgent determines it is in a blocking situation due to a very high traffic volume, it changes its behavior by entering *selfish* mode. In this example, the IntersectionAgent in zone “A” determines that all the vehicles that are entering the intersection are blocked. Because of this, it enters *selfish* mode and stops using the information received from the nearby IntersectionAgents (in this case, the agent in zone “B”).
  The actions performed by the agent are: Ir requests the IntersectionAgent in “B” to limit the vertical traffic flow (the IntersectionAgent in “B” will acknowledge this request because it is in *altruistic* mode yet), and then it sends the new phases configuration to the TLAgents in the intersection.
3. **Zone “A” normal flow** (Figure 3c): The decisions taken by the IntersectionAgent in the previous case have been successful, and, therefore, the congestion situation is over. This allows the IntersectionAgent in zone “A” to change back its behavior to *altruistic* mode. Now, the vertical traffic flow in the zone “B” is higher than before. This is because of the traffic light phase duration changes performed in “B” to help the IntersectionAgent in “A”. Once the blocking state is solved, the phases duration can be reverted to the best possible values for intersection “B”.

## 5. Results

In this section, we are going to define the experiments carried out for evaluating the proposals of Section 3 and Section 4.

Due to the high complexity of validating this kind of proposals in real-life scenarios, we have chosen to use a microscopic traffic simulator (SUMO [42]). This type of tool allows us to reproduce a scenario with conditions close to those expected from real scenarios, but with a very low cost.

### 5.1. Simulation Scenario

To carry out experiments, we have chosen a realistic simulation model, as it will provide better and more reliable results. Specifically, we have chosen the scenario called “Travel and Activity PAtterns Simulation (TAPAS) Cologne” [43,44] that is referenced in the SUMO documentation. It is a complete simulation scenario of the German city of Cologne. It was created by the Institute of Transportation Systems at the German Aerospace Center (TIS-DLR), and its goal is to reproduce, with the maximum possible realism, the urban traffic of Cologne. It defines a map of 400 km${}^{2}$ and 24 h of traffic.

The original simulation scenario is composed of a road network with 68,642 edges, 30,354 nodes and 1,547,333 routes, which causes very large simulation times. This is very inconvenient for the experiments that we have carried out, and, therefore, we have decided to crop the scenario in a smaller portion, still representative of the original one, but presenting lower simulation times. To obtain this sub-scenario, we have analyzed the features of the network used in [45], where they test a simulated network of the Lower Downtown Toronto.

Our sub-scenario is composed by 1416 edges and 716 nodes (from which 73 are intersections managed by traffic lights, and the rest are managed by priority rules). The routes modelling the traffic of the scenario have also been reduced to a more manageable number. We have selected the routes that are related to the cropped sub-scenario map, 246,374 in total.

**Figure 4:**
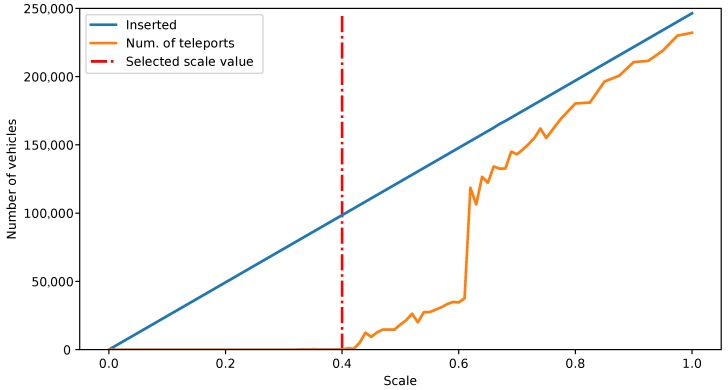
Evolution of the teleports number according to the scale parameter.

According to the “TAPAS Cologne” scenario documentation, the traffic inserted in the simulation should be scaled for the model to work correctly. This can be configured by setting the parameter *scale*, and the recommended value is 0.3. In our case, as we are working with a sub-scenario of the full “TAPAS Cologne”, we must recalculate this recommended value. We have carried out a set of simulations while increasing the *scale* parameter by 0.01 until we start seeing teleported vehicles in the simulation (teleporting is an event defined by SUMO, in which vehicles are taken out of the simulation and inserted again later to avoid the simulation blocking due to a too high traffic rate [46]). Figure 4 show the results of the *scale* value calculation.

The results of these simulations have led us to decide to use a 0.4 value for the *scale* parameter. This is the value where the number of teleported vehicles starts rising, as Figure 4 shows. The specific value for teleports is 241 for that scale, which is still a reasonable low value and allows us to insert the highest possible volume of traffic without compromising the realism of the simulation results. Scaling the traffic volume by 0.4 means that the actual number of vehicles and routes inserted will be of 98,550.

**Figure 5:**
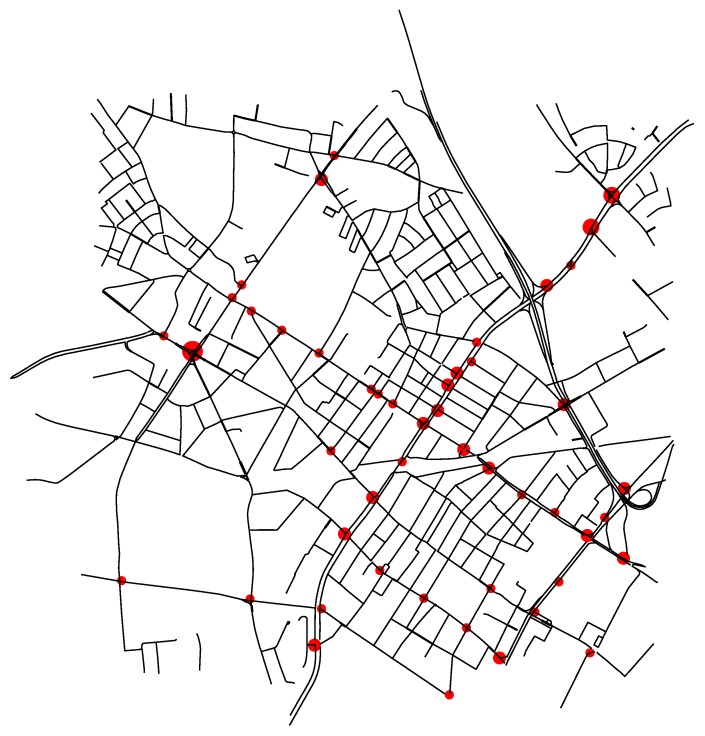
Representation of network edges (black lines) and intersection agents (red dots).

Once the sub-scenario has been selected, we have integrated it into the MAS. In Figure 5 we show the roads of the sub-scenario (in black lines) and the IntersectionAgents (red dots). The size of the dots indicates the number of TLAgents managed by each IntersectionAgent.

### 5.2. Sensor Network Optimization Experiments

We have defined a set of SUMO simulations to evaluate the proposal of the Section 3.2. As a traffic scenario, we have used the sub-scenario defined in Section 5.1. These simulations are carried out to determine the maximum value of data that can be obtained by inserting N camera sensors in the network, and then compare it with the values obtained when we place the cameras in the edges indicated by the centrality lists and with the proposed location system. The definition of experiments we have carried out is based on the proposal in [17]. In this work, the authors compare their proposal with two location methods called Random Road Location Method (RRLM) and Arterial Road Location Method (ARLM). RRLM consists of selecting random locations in the network and repeating the experiments 10 times, using the mean values obtained. In ARLM, sensors are located in the roads depending on the priority degree of those roads. For roads with the same priority, the sensor location is also random, following the RRLM process. In our case, we only include the random selection of locations, as our scenario is a realistic city network where most of the streets share similar features (maximum velocity, length, etc.), which leads the ARLM system to become an RRLM in practice. Following this scheme, we define four experiments:

- ***Max. value (Simulation data)***: A simulation is carried out, and the position of every vehicle is stored in each simulation step (setting simulation step to 1s). If an edge is longer than 120 m, the edge is divided in sub-edges, so the limit of the camera vision is not surpassed. We use a counter for each edge (or sub-edge), and it is incremented each time there is a vehicle located in it. At the end of the simulation, we get a list of edges ordered by the occupation level.
- ***Random locations***: In this experiment, we have simulated the results of applying a random location selection for sensors. We have selected from 1 to 32 random locations, and then we have obtained the results of the simulation. This process has been repeated 10 times, choosing new random locations each time. Once finished, the resultant values are the result of calculating the average value for the 10 repetitions.
- ***Betweenness centrality***: Using the SUMO libraries, the network is loaded in the computer memory and then transformed into a graph using the NetworkX library. This directed graph is then transformed into a line-graph and then the “betweenness_centrality” is applied. Using the resulting list, we carry out a set of simulations where we place from 1 to 32 cameras in the places indicated by the first positions of the list. Then, we store the number of vehicles that were counted by the cameras.
- ***Proposed system***: The process followed in order to obtain the betweenness centrality list is repeated. Then, we create a list with historical traffic data (we use the results of a previous simulation) and then we generate a list that combines both using an α factor equal to 0.5.

**Figure 6:**
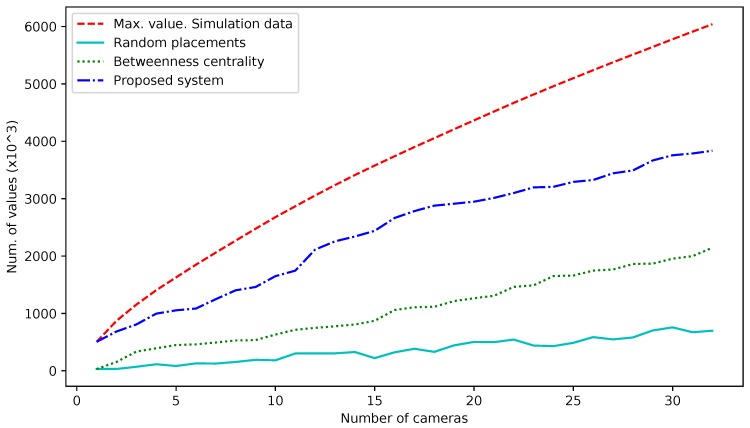
Number of values counted by camera sensors.

In Figure 6, the obtained results are shown after carrying out the four experiments. The *x*-axis represents the increment on the number of cameras used in the network, while the *y*-axis represents the number of values measured for each camera in each experiment. The red intermittent line shows the maximum theoretical value in case the cameras are on the edges with the highest occupation.

### 5.3. MAS Traffic Light Management Experiments

The experiments shown in this section have as main goals to measure the improvement of the system in terms of trip duration through the intelligent management of traffic lights, and to determine the number of messages generated by the sensors to feed each possible solution, in order to assess the improvement in each proposal (that is, using only the MAS or using the MAS with the optimized sensor network).

Using the set of traffic data shown in Section 5.1, we have defined and carried out a set of experiments to measure the trip duration of each vehicle and the number of messages generated by the sensors. We have used different system configurations in each case:

- ***Static phases***: In this experiment, we use the default TAPAS scenario configuration. The TAPAS traffic network defines a set of static phases for traffic lights in each one of its intersections. These phases do not change during the simulations. There are no sensors deployed in the network.
- ***Actuated traffic lights***: This kind of traffic lights management is based on the location of sensors in a fixed distance to intersections. The traffic lights phases change depending on the information received from these sensors. We use in this experiment the SUMO traffic simulator actuated traffic lights feature, included among the network configuration possibilities. In this case, there are induction loop sensors deployed in the network at a fixed distance from each intersection controlled by traffic lights. These sensors send a message each time a vehicle activates them by passing over.
- ***Proposed MAS***: We integrate the proposed MAS into SUMO to change the traffic lights phases dynamically during the simulation. When a camera sensor is deployed, it monitors the vehicles in its coverage area. If traffic jams are detected in that area, it sends a message. In addition, every vehicle in the system emulates a GPS sensor. If the velocity of the vehicle is less than half of the minimum between its maximum velocity and the maximum velocity of the road, it sends a message, notifying the situation. Note that, when the vehicle is in the coverage area of a camera sensor, it will not notify the situation, relying on the camera information.

**Figure 7:**
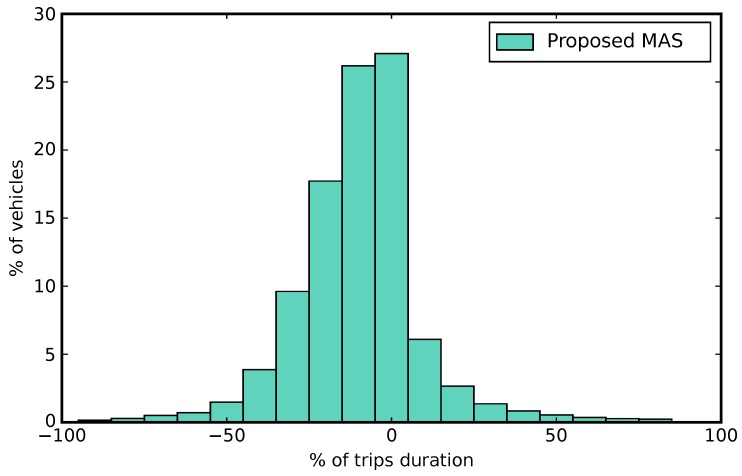
Percentage of increase or decrease in the trip duration over the percentage of vehicles.

In Figure 7, we show the results of comparing the “Static phases” experiment with the “Proposed MAS” experiment . The *x*-axis represents the percentage of increase or decrease in the trip duration (using steps of 10%) and the *y*-axis represents the percentage of vehicles inserted over the total vehicles in the simulation.

**Figure 8:**
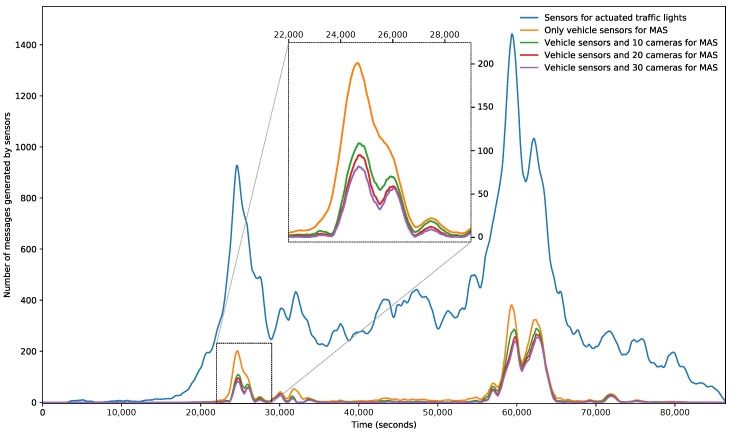
Number of messages generated by the sensor network during the simulation time.

The results of counting the number of messages generated by the sensor network in each experiment are shown in Figure 8. The *x*-axis represents the total simulation time (between 0 and 86,400 s: 24 h) and the *y*-axis shows the total number of messages generated in the system in each instant of time. To facilitate the visibility of the figure, we have zoomed the results between 22,000 and 29,000 s.

## 6. Discussion

We are starting the section commenting on the results obtained in Section 5.2. Those experiments had as a goal to verify the viability of the methodology used for the selection of the sensor location in the traffic network and measure the obtained results. In Figure 6, we show the values obtained when we insert into the simulation from 1 to 32 camera sensors. These sensors can count vehicles inside of his vision range (a maximum of 120 m in front of the camera location).

We can see how, from the results from using the ordered list based in the network centrality (dotted green line), we obtain a curve whose slope is lower than the slope from the curve representing the theoretical maximum value (intermittent red line) but with a constant increment.

The most important comparison between the experimental results is the one with the random road location method (RRLM), which follows the methodology proposed in [17]. Comparing the results of our proposal with the results obtained in the RRLM experiment, the improvement of using a centrality-based selection versus a random selection is shown. In addition, the results of the centrality experiment can be considered favorable, given that only the network topology has been used in this case to decide the location of the cameras. We can conclude that these results show how the use of centrality calculations is useful for this kind of environment.

The list obtained with our proposed methodology offers better results than the list obtained only using the centrality calculation, and also present an improvement margin by adjusting the correction factor α, which can be used to adapt the results depending on the reliability of the historical data used.

Once the sensor network has been defined and the sensors have been distributed along it for the support of the MAS, we have carried out the experiments described in Section 5.3.

We have measured the trip duration for each vehicle, and then we have compared the times obtained using the dynamic traffic lights management systems (that is, the actuated traffic lights and our proposal) with the trip duration obtained when using the static traffic light phases. The results of the comparison can be summarized by measuring the number of vehicles whose trip duration is reduced when compared with a static traffic lights scenario, the number of vehicles that do not see their trip duration modified, and, finally, the number of vehicles that show an increase in trip duration. These measurements can be performed both for the actuated traffic lights system and our proposal, and they are shown in Table 2 in percentages.

The table shows that not every vehicle has improved its trip duration when compared with the static traffic lights. The explanation for this can be found in the behavior of the dynamic systems, where some traffic flows will always be prioritized over others, causing an increase in waiting time for some vehicles. Nevertheless, the number of vehicles suffering for this increment is low (below 13% in both cases), and the time increase for half of those vehicles is below 10%. Therefore, it is possible to say that the dynamic change of traffic light phases offers a global improvement to the scenario in terms of trip duration.

If we compare the results between the actuated traffic lights and our proposal, it is true that the improvement of using the MAS might not seem too remarkable to justify using one system over the other, but there are some considerations that would indicate that our proposal is still better than the other one: first, the actuated traffic lights system depends on installing an induction loop in every edge of the traffic network that is attached to a traffic light managed intersection, while our proposal depends only on the optimized location of sensors across the network, which would noticeably decrease the overall infrastructure cost. Finally, the use of MAS provides a collaborative platform for the achievement of global goals across a traffic network, which cannot be obtained from isolated intersection management of actuated traffic lights.

From the generated messages volume point of view, and, using the results from Figure 8, we can obtain the following conclusions. First, we can observe how, around the time intervals centered in 25,000 and 60,000 s, there is a very noticeable increment of number of exchanged messages. These intervals of time correspond with peaks in the volume of traffic flows in the simulation scenario. As in the actuated traffic lights system (blue line in the figure), there is one message of each vehicle that passes over the sensors (induction loops), and the system generates a high volume of messages during all the simulation period, independently of the real traffic status (this system is not able to distinguish between congestion and not congestion states). On the other hand, we can see that the peaks of number of messages generated in the experiments based in the MAS (every other line in the figure) are centered only around the areas with peaks of traffic flows.

Regarding the possible improvements of using an optimized sensor network additionally to the MAS, we can observe in the zoomed section of Figure 8 how using the MAS without an optimized network (orange line) shows the maximum number of messages generated. When the experiment is repeated using an optimized network with 10 camera sensors positioned by following the proposed methodology (green line), it is possible to perceive a significant reduction in the generated messages quantity (specifically, there is a reduction of 47.4% or 216,860 messages less over the previous results). When more sensors are located in the network (red and purple lines), the number of messages exchanged is still being reduced, but with a lower effect. This result was predictable, as the first cameras are already positioned in the more relevant positions of the network. As a conclusion, the amount of data exchanged in the network needed for the decision-making process will be lower in the MAS, which will also reduce the communications overhead.

One of the most difficult situations to solve in traffic congestion states is the blocking of some vehicles in the intersections, where they stay stopped for very long periods of time. This kind of event has been reproduced during our experiments, and we have observed that the MAS is able to react specifically to avoid them. If we compare the results between experiments, we can conclude that 1790 vehicles have been “unlocked” by the MAS, and, consequently, their trip duration has been lowered. The reaction of agents to these blocking situations can also be considered an important advantage of MAS over actuated traffic light systems.

The proposal of this paper was to optimize a sensor network through the study of the traffic network topology and then use the information provided by this network to intelligently manage the traffic lights using an MAS.

After analyzing the results, we can conclude that the election of the locations of the sensors using centrality measurements from a graph provides useful and reliable information to be used in ITS applications. When compared with some of the solutions shown in Section 2 related to the positioning of sensors in vehicular traffic networks, one of the most relevant advantages of using our proposal is the low dependency from having historical data of traffic flows for it to work. The study based on the centrality of the network has shown that, using only the network topology, it is possible to find near-optimal sensor positions, where the sensors are able to provide a high volume of relevant information, while reducing the number of necessary message exchanges in the MAS.

Furthermore, when comparing the MAS results with other solutions like the static definition of traffic light scheduling or the dynamic management of traffic lights phases (actuated traffic lights system), we show that the use of our MAS is effective for the reduction of trip duration in vehicles.

The goals defined in Section 1 have been covered in the following way: demonstrating that the betweenness centrality is a valid measure for the selection of relevant zones in a traffic sensor network, validating the agent behavior proposed to modify the traffic lights phases and obtain a reduction in trip duration, and, finally, developing the needed tools for the model of sensors and agents in a microscopic traffic simulator such as SUMO.

## Figures and Tables

**Table 1:** Intelligent Transportation Systems’ sensor classification.

Sensors equipped in vehicles	Global navigation Satellite System (GNSS)	GPS and Galileo are the most well-known systems of this type. They provide information about the position and current velocity of the vehicles.
Autonomous sensors	In-roadway sensors	Senses the presence of a conductive metal object by inducing currents in the object, which reduce the loop inductance.
		Magnetic sensors: Similar to the inductive-loop detectors, are able to sense the presence of vehicles using the perturbation they cause in the magnetic field.
	Over-roadway sensors	Microwave or laser radar sensors: Detect vehicles by transmitting microwave or laser signals and receiving the echoes from them.
		Infrared sensors: Detect vehicles by receiving the energy emitted by roadways and vehicles or energy reflected from them.
		Cameras: It is possible to detect vehicles by the processing of the images taken by one or more video cameras.

**Table 2:** Results summary (Percentage of total vehicles).

Trip duration	Lower	Equal	Higher
Actuated Traffic Lights	58.70	28.41	12.89
Proposed Multi-Agent System	60.52	27.08	12.41

## Abbreviations

The following abbreviations are used in this manuscript:

ACTAM	Adaptive and Cooperative Traffic light Agent Model
ARLM	Arterial Road Location Method
ATMS	Advanced Traffic Management Systems
EA	Evolutionary Algorithms
GA	Genetic Algorithm
GNSS	Global Navigation Satellite System
GPS	Global Positioning System
ITS	Intelligent Transportation Systems
MAS	Multi-Agent Systems
NSLP	Network Sensor Location Problem
O-D	Origin-Destination
PSO	Particle Swarm Optimization
RRLM	Random Road Location Method
SUMO	Simulation of Urban MObility
TAPAS	Travel and Activity PAtterns Simulation
TCP	Transmission Control Protocol
TIS-DLR	Institute of Transportation Systems at the German Aerospace Center
TraCI	Traffic Control Interface
WSN	Wireless Sensor Networks
